# Dentigerous Cyst or Adenomatoid Odontogenic Tumor: Clinical Radiological and Histopathological Dilemma

**DOI:** 10.1155/2014/514720

**Published:** 2014-07-01

**Authors:** Shivesh Acharya, Ashima Goyal, Vidya Rattan, Kim Vaiphei, Sarabjot Kaur Bhatia

**Affiliations:** ^1^Department of Pediatric and Preventive Dentistry, Oral Health Sciences Center, Postgraduate Institute of Medical Education and Research, Chandigarh 160012, India; ^2^Department of Oral and Maxillofacial Surgery, Oral Health Sciences Center, Postgraduate Institute of Medical Education and Research, Chandigarh 160012, India; ^3^Department of Histopathology, Postgraduate Institute of Medical Education and Research, Chandigarh 160012, India

## Abstract

Adenomatoid odontogenic tumor (AOT) is a well-recognised slow growing benign tumor derived from complex system of dental lamina or its remnants. This lesion is categorised into three variants of which the more common variant is follicular type which is often mistaken for dentigerous cyst. We present a case of AOT in a 14-year-old male who was misdiagnosed as dentigerous cyst. Clinical radiological and therapeutic characteristics of the case are commented on in detail.

## 1. Introduction

Adenomatoid odontogenic tumor (AOT) was first described by Ghosh in 1934 [1] as an adamantinoma of the maxilla and was first recognised as distinct entity by Stafne in 1948 [2]. Later on it has been described under various names like adenoameloblastoma, cystic complex composite odontoma, ameloblastic odontogenic tumor, odontogenic adenomatoid tumor, and so forth. WHO in 1971 adopted the term proposed by Philipsen and Birn [3] as AOT and defined lesion as “a tumor of odontogenic epithelium with duct-like structures and with varying degrees of inductive change in the connective tissue. The tumor may be partially cystic, and in some cases solid lesion may be present as masses in the wall of large cyst. It is believed that lesion is not a neoplasm” [4]. Philipsen et al. subdivided this condition into three groups referred to as follicular, extrafollicular, and peripheral. These variants have common histologic characteristics that indicate a common origin as derived from the complex system of dental lamina or its remnant [5]. The follicular and extrafollicular variants account for 96% of all AOT and of these 71% are follicular variants. The peripheral variant is the rarest with only 18 cases reported so far [6]. The follicular variant is predominantly associated with the crown and often part of the root of an impacted (unerupted) tooth. The most frequently associated tooth is the maxillary canine rarely the permanent molars. Based on the clinical and radiographic examination follicular variant is often initially mistaken as dentigerous cyst [3]. Here we present a case of AOT which presented as cyst like lesion around the crown of unerupted maxillary canine and was initially mistaken as dentigerous cyst.

## 2. Case Report

**Figure 1:**
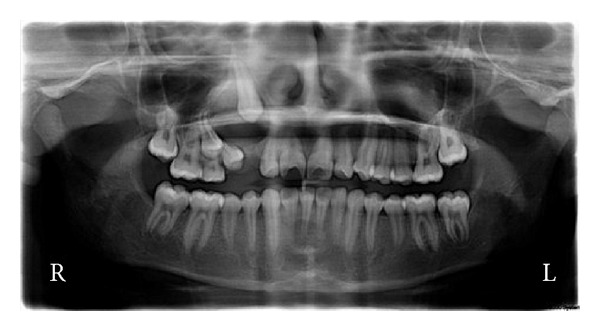
Preoperative orthopantomograph showing radiolucent lesion in relation to unerupted right maxillary canine.

A 14-year-old boy presented to the Unit of Pedodontics and Preventive Dentistry, Oral Health Sciences Centre, PGIMER, Chandigarh, India, with swelling and pain on right side of the face. The detailed history reported by the father revealed that they first noticed the swelling 4-5 months back. The swelling was progressively increasing in size and straw-colored fluid occasionally exuded from the swelling. A private dental practitioner was consulted for the same who extracted maxillary right primary canine and first molar. The diagnosis of infected cyst (dentigerous) was made histopathologically. Swelling persisted one month after treatment so the patient was referred to Oral Health Sciences Centre, PGIMER, Chandigarh. At clinical examination on initial visit an extraoral facial swelling approximately measuring 1 × 3 cm was noted on right side of the face obliterating the nasolabial fold. Swelling was tender and fluctuant with well-defined margins. Intraoral examination revealed permanent dentition with missing permanent canine, first and second premolar, and swelling extending from right maxillary lateral incisor to first premolar region. Orthopantomograph revealed a well-defined radiolucent lesion in relation to unerupted maxillary right permanent canine, first premolar, and second premolar, extending from distal surface of lateral incisor to mesial surface of first premolar and which has also caused the displacement of roots of the adjacent lateral incisor and the first premolar (Figure 1).

**Figure 2:**
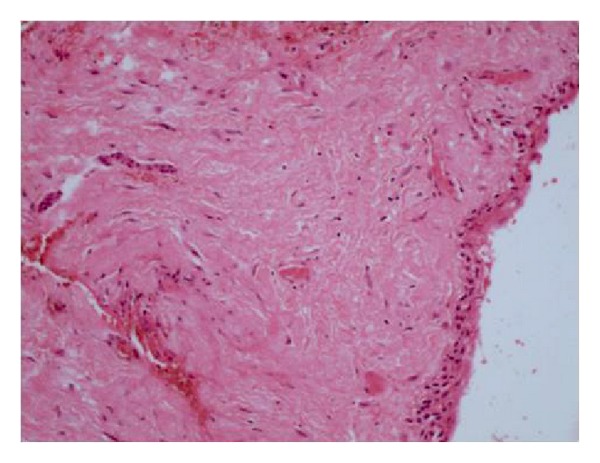
Focus showing fragments lined by squamous epithelium with wall composed of fibrocollagenous tissue consistent with dentigerous cyst (hematoxylin and eosin 10x).

On diagnostic aspiration, straw-colored fluid was drawn from the lesion. Based on clinical and radiographic evaluation a provisional diagnosis of dentigerous cyst was made and conservative approach was planned to marsupialize the cystic swelling. Swelling was marsupialized under local anesthesia and an acrylic stent was positioned to maintain patency and to allow for eruption of permanent canine. Part of cystic lining was evaluated histopathologically which showed fragments lined by squamous epithelium with wall composed of fibrocollagenous tissue with minimum inflammation features consistent with dentigerous cyst (Figure 2).

**Figure 3:**
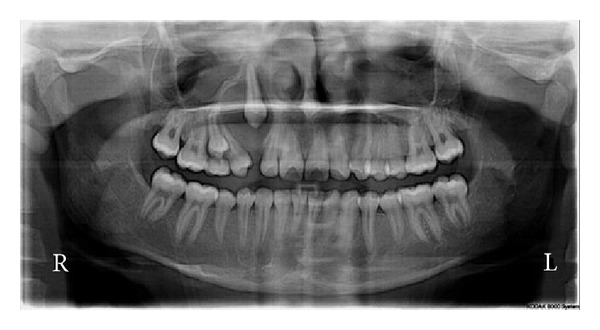
Panoramic radiograph taken after marsupialization of the cyst showing no movement of the canine.

The patient was put on antibiotics and analgesics for 5 days and was followed up on monthly basis. The patient was reinforced to maintain oral hygiene on each follow-up and was evaluated for eruption of canine radiographically. At 2-month follow-up visit, occasional discharge from the swelling was present and detailed evaluation of panoramic radiograph (Figure 3) showed no eruptive movement in canine and radiolucent area was still persisting.

**Figure 4:**
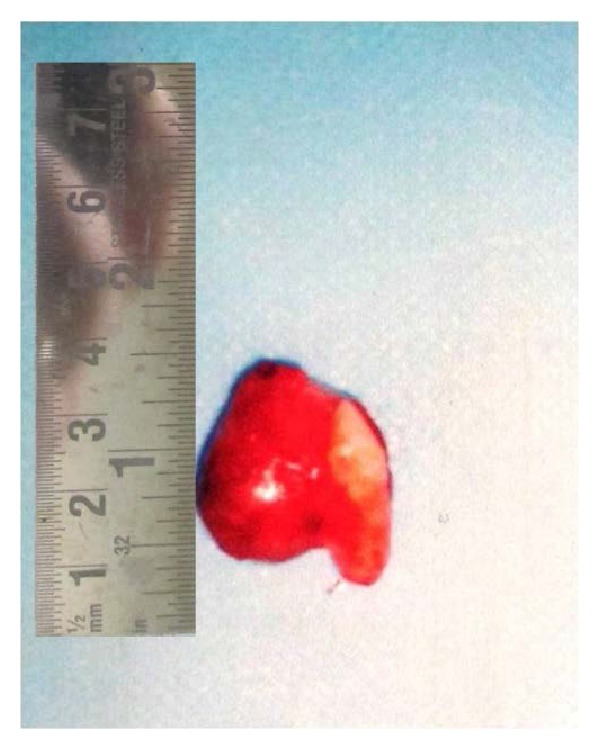
Canine along with the lesion in toto removed surgically.

At this stage a decision to surgically remove the canine along with removal of the lesion in toto was taken. The lesion was completely enucleated under local anaesthesia along with permanent canine. The cyst was separated easily from the adjoining bone and there was no evidence of oronasal and oroantral communication and the palatal mucosa was intact. The wound was then sutured closed. The surgical specimen enveloping a permanent tooth was smooth and reddish in color and measured approximately 20 × 20 × 15 mm (Figure 4).

**Figure 5:**
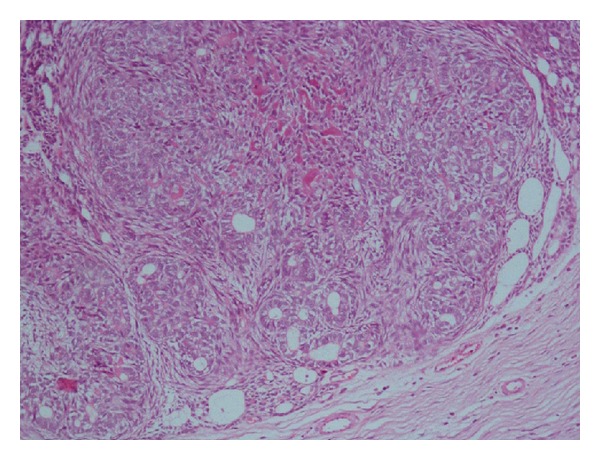
Solid proliferation of polygonal and spindle shaped cells with only scanty stroma of connective tissue associated with duct-like and rosette-like structures suggestive of AOT (hematoxylin and eosin stain 40x).

The surgical specimen was submitted for histopathological examination. Histopathology report revealed solid proliferation of polygonal and spindle shaped cells with only scanty stroma of connective tissue associated with duct-like and rosette-like structures. Deposition of eosinophilic homogenous material within the rosette-like structures was also seen. (Figure 5).

**Figure 6:**
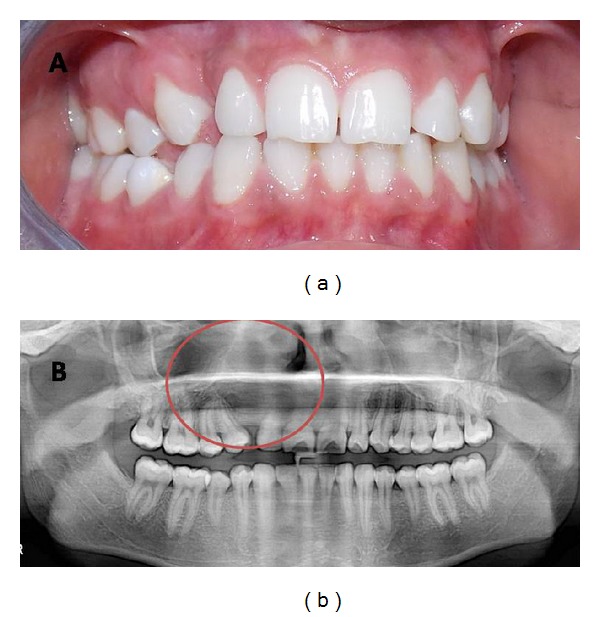
(a) Clinical presentation at 3-year follow-up. (b) Orthopantomograph taken at three- year follow-up.

Patient was followed up regularly and no evidence of any discharge or recurrence of swelling was noted. Orthopantomograph taken at three-month follow-up showed sign of resolution of radiolucency. The patient was advised to undergo multibracketed treatment for space closure. At three-year follow-up, there was no evidence of any discharge or recurrence (Figure 6(a)) and panoramic radiograph revealed normal bone healing (Figure 6(b)).

## 3. Discussion

An extensive review of 500 cases of AOT has been conducted by Philipsen et al. [6]. Leon et al. described a multicentre study of both the clinicopathological and immunohistochemical features of 39 cases of AOT. Two-third of these were diagnosed in the second decade of life, and over 50% occurred in adolescents between ages of 13 and 19 [14].

Our patient falls into this group, but it is noted that range of occurrence is very wide (3–82 years). Both follicular and extrafollicular variants occur more commonly in the maxilla than in the mandible, with a ratio of 2.1 : 1. The female : male ratio for all age groups and AOT variants together is 2 : 1, with an even higher female preponderance (approximately 3 : 1) among certain Asian populations [5, 14]. Our patient is an Asian male. Cystic presentation of AOT has been reported way back in 1915 by Harbitz who reported the lesion as “cystic Adamantoma” [15]. The most common presentation of AOT radiologically is the unilocular cystic mass enclosing the unerupted tooth (the reason it is commonly taken as a dentigerous cyst). Also, histopathologically, the lesion may rarely present with a cystic component. Only recently the cystic nature of AOT has been in debate. The bisected lesion may show varying degrees of cystic change and rarely the tumor may entirely be cystic [16]. The systematic review of the literature of AOTs associated with or originating from an odontogenic cyst has been conducted by Gadewar et al. [16]. The cystic component of AOT has been variedly termed as dentigerous cyst [9, 10, 13], calcifying odontogenic cyst [17, 18], or unilocular ameloblastoma [19]. However, in pediatric population very few cases have been described that arise in association with a dentigerous cyst. A systematic search of the English language medical literature revealed only seven such cases in children and adolescents in the age range of 8–18 years (PubMed search using the key words adenomatoid odontogenic tumour, dentigerous cyst). The clinical characteristics of these cases and the current case are summarised in Table 1.

It is noted that the male to female ratio is 7 : 1 and nearly all the cases occurred during the second decade of life except one which is reported in 8-year-old male child. Most of the lesions appeared as a well-circumscribed unilocular radiolucency around the unerupted tooth and the most commonly involved tooth was the maxillary canine (6 cases).

The origin of the AOT is controversial. Some have focused on the idea that its origin is from the odontogenic epithelium of the dentigerous cyst, while others believe that tumors could be derived from epithelial remnants of the dental lamina complex system. The lesion grows into a nearby dental follicle or next to follicle leading to “envelopmental” theory [20]. Chen et al. even suggested the term “hybrid variant” where AOT is derived from dentigerous cyst. In our case the tumor surrounded the fully formed canine suggesting an envelopmental pathogenesis or “hybrid variant” [12].

The interest and relevance of the present case are the difficulty to diagnose accurately based on the radiograph and histopathology. The initial histopathological report in the present case stated findings suggestive of dentigerous cyst and later report suggested findings corresponding to those of adenomatoid odontogenic tumor. Whether it was dentigerous cyst transforming to adenomatoid tumor or a cystic variant of adenomatoid odontogenic tumor in the present case could not be stated with exactitude as initially to preserve associated tooth only part of cystic lining was removed for histopathological evaluation. Gadewar et al. [16] suggested that incisional biopsy depicting the cystic lining alone would inaccurately identify the lesion as dentigerous cyst or unicystic ameloblastoma. The use of MRI and particularly dynamic contrast enhanced MRI to distinguish AOT from other odontogenic lesions that have been described [21].

Both dentigerous cyst and adenomatoid odontogenic tumors are entirely benign, encapsulated lesions, and enucleation poses no major difficulties. If the dental follicle is found to be uninvolved during surgery and if it can be easily separated from the tumor, it may be possible to remove the lesion while leaving the teeth in place [22]. In the present case report permanent canine was embedded in the tumor, and the large size and close approximation of the lesion to the erupted teeth made it impossible to save the tooth. No aggressive behaviour on the part of the adenomatoid tumors has been described, and recurrence is very rare following correct enucleation of the primary lesion [23].

## 4. Conclusion

As depicted in the present case, AOT is often mistaken as dentigerous cyst radiologically as well as histopathologically, and in that context even in pediatric population few case reports of AOT arising from or associated with dentigerous cyst have been reported. However, the present case highlights the importance of the fact that in cases of unilocular lesion surrounding the impacted tooth in the anterior maxillary region the treatment as per AOT should be followed.

## Figures and Tables

**Table 1:** Clinical data of the reported cases of adenomatoid odontogenic tumor (AOT) arising from a dentigerous cyst in children and adolescents.

Reference	Age/Sex	Race	Radiographic	Features	Site
Valderrama [7]	16 females	Filipino	Unilocular radiolucency	14 crown surrounded	Maxilla
Warter et al. [8]	8 males	Nigerian	Unilocular radiolucency	13 crown surrounded	Maxilla
Tajima et al. [9]	15 males	Japanese	A well-defined radiopaque mass	Crown of unerupted 28	Maxillary sinus
Garcia-pola et al. [10]	12 males	Spanish	Unilocular radiolucency	23 crown	Maxilla
Bravo et al. [11]	14 males	Not stated	Unilocular radiolucency	23 crown surrounded	Maxilla
Chen et al. [12]	18 males	Chinese	Unilocular radiolucency	23 crown surrounded	Maxilla
Nonaka et al. [13]	13 males	Not stated	Unilocular radiolucency	23 crown	Maxilla
Present case	14 males	Asian	Unilocular radiolucency	13 crown	Maxilla
